# Late Diagnosed Left Coronary to the Pulmonary Artery Large Fistulae: An Interesting and Incidental Cath Lab Finding

**DOI:** 10.1155/2016/1459607

**Published:** 2016-12-07

**Authors:** Marcos Danillo P. Oliveira, Pedro H. M. Craveiro de Melo, Érlon O. Abreu-Silva, Fernando Barbiero Coura, Gleyson Moraes Rios, Daniel Izzet Potério

**Affiliations:** Vitta in Core Intervenções Cardiovasculares, Santa Casa de Araras, São Paulo, SP, Brazil

## Abstract

Coronary artery anomalies are congenital changes in their origin, course, and/or structure. Most of them are discovered as incidental findings during coronary angiographic studies or at autopsies. A coronary artery fistulae involve a communication between a coronary artery and a chamber of the heart or any segment of the systemic or pulmonary circulation. We present herein the case of a 67-year-old man with a recent history of exertional angina and dyspnea to usual daily activities whose coronary angiogram revealed an interesting and incidental coronary-pulmonary artery large fistulae.

## 1. Introduction

Coronary artery anomalies (CAA) are a diverse group of congenital disorders, and the pathophysiological mechanisms and manifestations are highly variable. Several controversies remain in terms of its incidence, classification, screening, heredity, and treatment [1–5].

Coronary artery fistulas (CAF) are abnormal communications between a coronary artery and a cardiac chamber or a major vessel [6]. They may be congenital or acquired due to trauma or iatrogenic causes [7].

Most of them are small, do not cause any symptoms, and are clinically undetectable. However, the larger CAF can cause coronary steal phenomenon, which leads to myocardial ischemia [8].

We report the case of a 67-year-old man with a recent history of exertional angina and dyspnea to usual daily activities whose coronary angiogram revealed severe and complex coronary disease in association with interesting coronary-pulmonary artery large fistulae incidentally diagnosed during the same procedure.

## 2. Case Report

**Figure 1:**
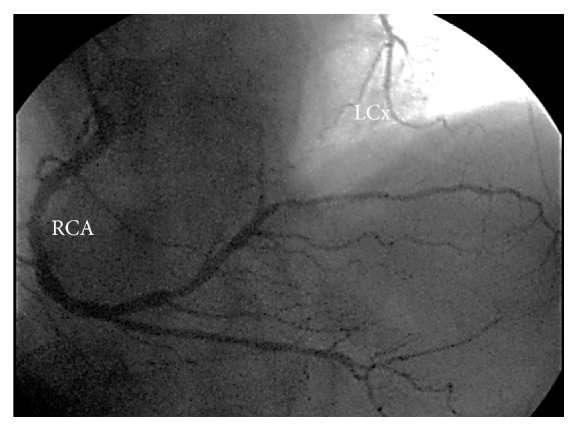
Selective contrast injection in the dominant RCA, showing ectasias and diffuse discrete lesions and a moderate narrowing at the proximal portion of the right posterolateral branch, and the well developed collateral circulation to the LCx. RCA: right coronary artery; LCx: left circumflex.

**Figure 2:**
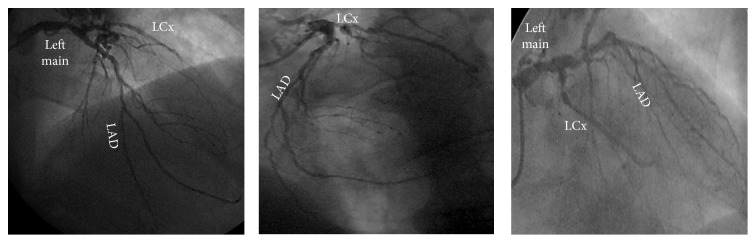
Selective contrast injection in the LCA showing the severe, calcified, and complex multisite obstructive coronary artery disease. LCA: left coronary artery; LAD: left anterior descending; LCx: left circumflex.

**Figure 3:**
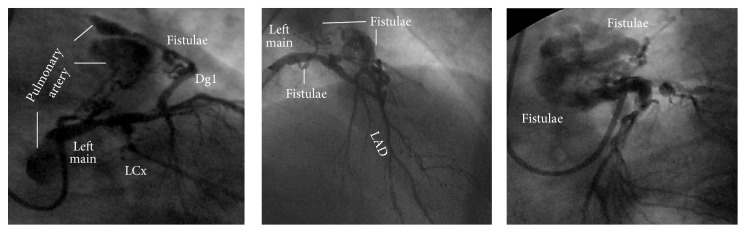
Selective contrast injection in the LCA showing the well developed fistulae, from the left main and, especially, the first diagonal branch to the pulmonary artery (retrogradely filled). LCA: left coronary artery; LAD: left anterior descending; LCx: left circumflex; Dg1: first diagonal branch.

A 67-year-old man, active, Caucasian, presented with a three months' history of exertional angina and dyspnea to usual daily activities. There were neither previous episodes of myocardial infarction, stroke, and coronary artery disease nor personal or familiar histories of sudden cardiac death. The resting electrocardiogram (ECG) showed sinus rhythm and left atrial and ventricular overload. The treadmill test showed an ischemic pattern and the coronary angiogram was, then, requested. The right coronary artery (RCA) showed a dominant pattern, with ectasias and diffuse discrete lesions and a moderate lesion at the proximal portion of the right posterolateral branch (Figure 1). The left coronary system, in turn, showed severe, calcified, and complex coronary artery disease: 50% at the shaft of the left main; diffuse narrowing lesions and subocclusion at the proximal portion of the left anterior descending; discrete lesions at the three diagonal branches; subocclusion at the ostium followed by total occlusion at the mid portion of the left circumflex, with a well developed collateral circulation from the RCA and discrete lesions at the first obtuse marginal branch (Figure 2). Surprisingly, a well developed fistula was found, from the left main and, especially, the first diagonal branch to the pulmonary artery (Figure 3). The left ventricle showed a hypertrophic pattern, with normal systolic function. There was no systolic aortic transvalvular gradient. Due to this association between severe and complex coronary artery disease and the large left coronary-pulmonary artery fistulae, the surgical intervention (bypass grafting plus ligation of the fistulae) was, then, proposed to the patient. He is, at the time of this report, waiting the call for the surgery, in the same functional status, without hospital readmissions or major adverse cardiac or cerebrovascular events.

## 3. Discussion

CAA are congenital changes in their origin, course, and/or structure. Several controversies remain in terms of its incidence, classification, screening, heredity, and treatment. Despite being mostly asymptomatic, clinical presentation in adults may result from myocardial ischemia, manifesting as angina, syncope, arrhythmias, and even sudden death. In young athletes, apparently healthy ones, they are the second most frequent cause of sudden death [1–5].

Most CAA are discovered as incidental findings during coronary angiographic study or at autopsy with incidence rate of 0.64% to 1.3% reported in the literature [1, 5].

CAF are abnormal communications between a coronary and a cardiac chamber or a major vessel [6]. They are usually congenital but may be consequent to trauma or iatrogenic causes [7].

The majority of CAF are small, do not cause any symptoms or hemodynamic compromise, and are clinically undetectable until echocardiography or coronary angiography is performed. They usually do not cause any complications and can spontaneously resolve. However, larger fistulae are usually three times the normal caliber of a coronary artery and may cause complications and/or symptoms, especially due to the coronary steal phenomenon, which leads to myocardium ischemia of the territory perfused by the related coronary artery. The mechanism is related to the diastolic pressure gradient and runoff from the coronary vasculature to a low-pressure receiving cavity. If the fistula is large, the intracoronary diastolic perfusion pressure progressively diminishes [7, 9].

Over time, the coronary artery leading to the fistulous tract progressively dilates, which, in turn, may progress to aneurysm formation, intimal ulceration or rupture, medial degeneration, atherosclerosis, calcification, mural thrombosis, and, rarely, rupture [7, 9].

The hemodynamic significance of the fistulous connection depends on the size of the communication, the resistance of the recipient chamber, and the potential for development of myocardial ischemia. High-output congestive heart failure has been described [7, 9].

Noninvasive 3D imaging of the coronary vasculature is advantageous. Traditionally, magnetic resonance imaging (MRI) has been a good alternative for imaging proximal coronary abnormalities, and newer imaging sequences have provided improved anatomic imaging as well as indices of coronary flow and function. Spatial resolution is often limiting, and the distal course and insertion of the fistulous connection may not be well imaged. Multidetector computed tomographic angiography (MDCTA) has provided excellent distal coronary artery and side branch imaging, with better temporal and spatial resolution than MRI. Several authors recommend MDCTA in imaging of coronary anomalies [10]. A retrospective study by Lim et al. [11], including 6341 patients, suggested that MDCTA is useful in detecting CAF, found in 56 patients (0.9%), a higher percentage than that generally found using conventional angiography. Moreover, MDCTA found CAF to lead most commonly to the pulmonary artery [11].

All interventional cardiologists and cardiac surgeons should be familiar with these anatomic variants since accurate recognition of the course and distribution of the coronary vessels are crucial for proper revascularization strategies in the presence of coronary artery disease [1, 5].

In our case, since there was an association between significant and complex multiple coronary lesions and a larger CAF, the surgical correction of both problems was considered as the best treatment option for the patient.
